# Time-Resolved Luminescence Properties of Laser-Fabricated Nano-diamonds

**DOI:** 10.1186/s11671-020-03393-y

**Published:** 2020-08-20

**Authors:** Juan Hao, Lingyun Pan, Minghui An, Yunzhi Dai, Bingrong Gao

**Affiliations:** 1 College of Electronic Science and Engineering, Jilin University, Changchun, 130012 People’s Republic of China; 2 College of Physics, Jilin University, Changchun, 130012 People’s Republic of China

**Keywords:** Nano-diamonds, Pulsed laser ablation, Luminescence, Surface modification

## Abstract

In the study, well-crystallized nano-diamonds with an average size of 3.8 nm are obtained via femtosecond laser ablation. Both steady-state and transient luminescence are observed. The luminescence peaks of nano-diamonds shift from 380 to 495 nm when the excitation wavelength changes from 280 to 420 nm. After passivation by polyethylene glycol-400N, the surface of nano-diamonds is significantly oxidized, which is verified by Raman and UV-Vis absorption spectra. Furthermore, there is no change in all the luminescence wavelengths, although the maximum intensity increases by 10 times. Time-resolved luminescence spectra reveal that trapping states can be modified by surface passivation, and this leads to stronger luminescence with a longer lifetime.

## Introduction

Carbon nanomaterials including quantum dots, nanowires, and thin films play an important role in the application of novel materials [1–6]. When compared with other carbon nanomaterials, nano-diamonds (NDs) exhibit unique advantages and applications in the electronics industry, genetic engineering, pharmaceutical engineering, and lithium-ion batteries [7, 8]. Zimmermann et al. demonstrated that heterostructure diodes built using ultra-small NDs exhibit high performance in the ultraviolet region as well as in terms of rectification and temperature stability at 1050 °C [9]. Wang et al. examined the delivery of epirubicin using NDs 2–20 nm in diameter, and this is expected to overcome chemoresistance in cancer stem cells and improve the therapeutic effect in hepatic cancers [10]. Ho et al. revealed that NDs only 2–5 nm in size can achieve gene delivery by entering the nucleus via passive transport [11]. Additionally, it shows that the performance of the battery can be significantly improved by adding NDs (< 5 nm) to the electrolytic cell of a lithium battery, and the effect is more positive with decreases in size [12]. Thus, it is imperative to produce NDs less than 5 nm in size.

However, it is difficult to fabricate uniformly distributed 5-nm NDs via conventional methods. Currently, the main method for preparing NDs is through a high temperature and pressure explosion. The size of NDs obtained by this method is about 4 nm, but the stand-by size always exceeds 100 nm due to agglomeration [13]. Although various de-agglomeration methods have been explored, even the best method can only disintegrate the large agglomerated particles to approximately 10 nm [14]. Extant studies continue to explore well-dispersed and ultra-small (< 5 nm) NDs and significantly more green, simple, and ambient methods. Sankaran et al. fabricated NDs in the range of 2–5 nm via microplasma dissociation of ethanol vapor [15]. However, ND chains are formed due to agglomeration [16]. Tan et al. successfully obtained well-dispersed NDs of 2–4 nm via femtosecond pulsed laser ablation in liquid (fs-PLAL) [17]. However, the conversion efficiency of graphite or carbon materials to diamond through ablation is excessively low to improve the yield of NDs [17–19].

Given the development of preparation technology, there is a gradual increase in studies on the optical properties of NDs. Shenderova et al. concluded that NDs include a series of luminescence centers from blue to red [20]. Schmidt et al. realized energy transfer and manipulation of the molecular chain via utilizing fluorescent NDs [21]. Furthermore, many biological applications were realized based on the luminescence properties of NDs via surface modification [22, 23]. Therefore, it is necessary to investigate their luminescence properties. In particular, luminescence dynamics constitutes helpful data to understand the mechanisms of emission process, and there is a paucity of studies examining the same.

In the study, we used fs-PLAL on bulk diamond in ethanol. As a result, a large amount of well-dispersed NDs with 3.8 nm average size and naked surface were successfully obtained. The NDs were then passivated by bio-compatible polyethylene glycol-400N (PEG_400N_). Both steady-state and transient luminescence of un-passivated and passivated NDs were investigated. The dynamics results clearly exhibit slower relaxation in passivated NDs when compared to un-passivated NDs, and this can explain the difference in their luminescence intensities.

## Methods

**Fig. 1:**
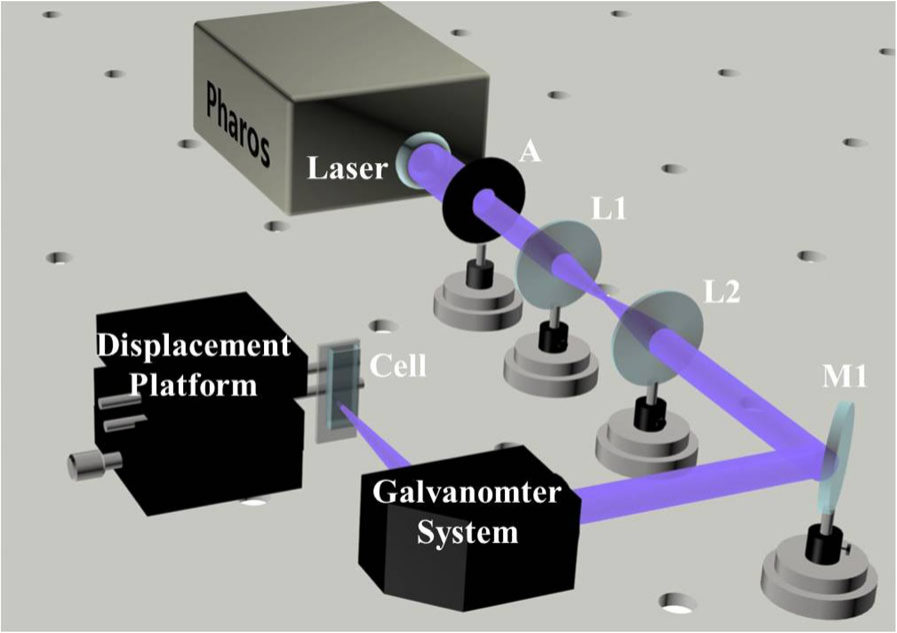
The experimental scheme for femtosecond laser ablation diamond bulk in ethanol

The custom-built laser ablation equipment is shown in Fig. 1. The femtosecond laser (Light-Conversion Pharos, 343 nm, ~ 300 fs, 90 kHz, 9.2 μJ∙pulse^−1^) first passed through aperture A1, then the beam expander (L1 and L2), and finally the galvanometer system (Sunny Technology, TSH8720M) composed of a F-theta system and field mirror aided by all-reflection mirror M1. Through the field mirror (*f* = 10 cm), the laser was focused on the solid-liquid interface of bulk diamond (2 mm in diameter, 1 mm in thickness) in a quartz cuvette (optical length = 10 mm) filled with ethanol. The focus scanned on the target in concentric circles 1 μm apart. After an hour, the colloid was collected and ultrasonicated for 45 min. Subsequently, the sample was divided into two parts wherein one was investigated without any treatment (termed as un-passivated NDs) and the other after surface passivation (termed as passivated NDs). For surface passivation, 0.1 ml PEG_400N_ (Sigma Aldrich, CAS: 25322-68-3) was dropped into a sample, and the mixture was subjected to ultrasonic treatment for 30 min, and then poured into the reactor. Finally, the reactor was maintained at 120 °C for 72 h in an incubator.

The morphology of NDs was characterized via a transmission electron microscope (TEM) and high-resolution transmission electron microscope (HRTEM) on the JEOL JEM-2100F. The size distribution was analyzed by the Nano Measurer software. Raman spectroscopy and Fourier transform infrared spectroscopy (FTIR) were performed to confirm the phase structure and surface ligands of NDs, respectively. Specifically, ultraviolet-visible (UV-Vis) absorption spectra and luminescence spectra were obtained to investigate the optical properties. The details of all the characterization equipment have been introduced in a previous study [24]. Time-resolved spectra were obtained using a fluorescence transient spectrometer (HORIBA, IHR320, 55ps) with a 390-nm NanoLED source (N-390).

## Results and Discussion

**Fig. 2:**
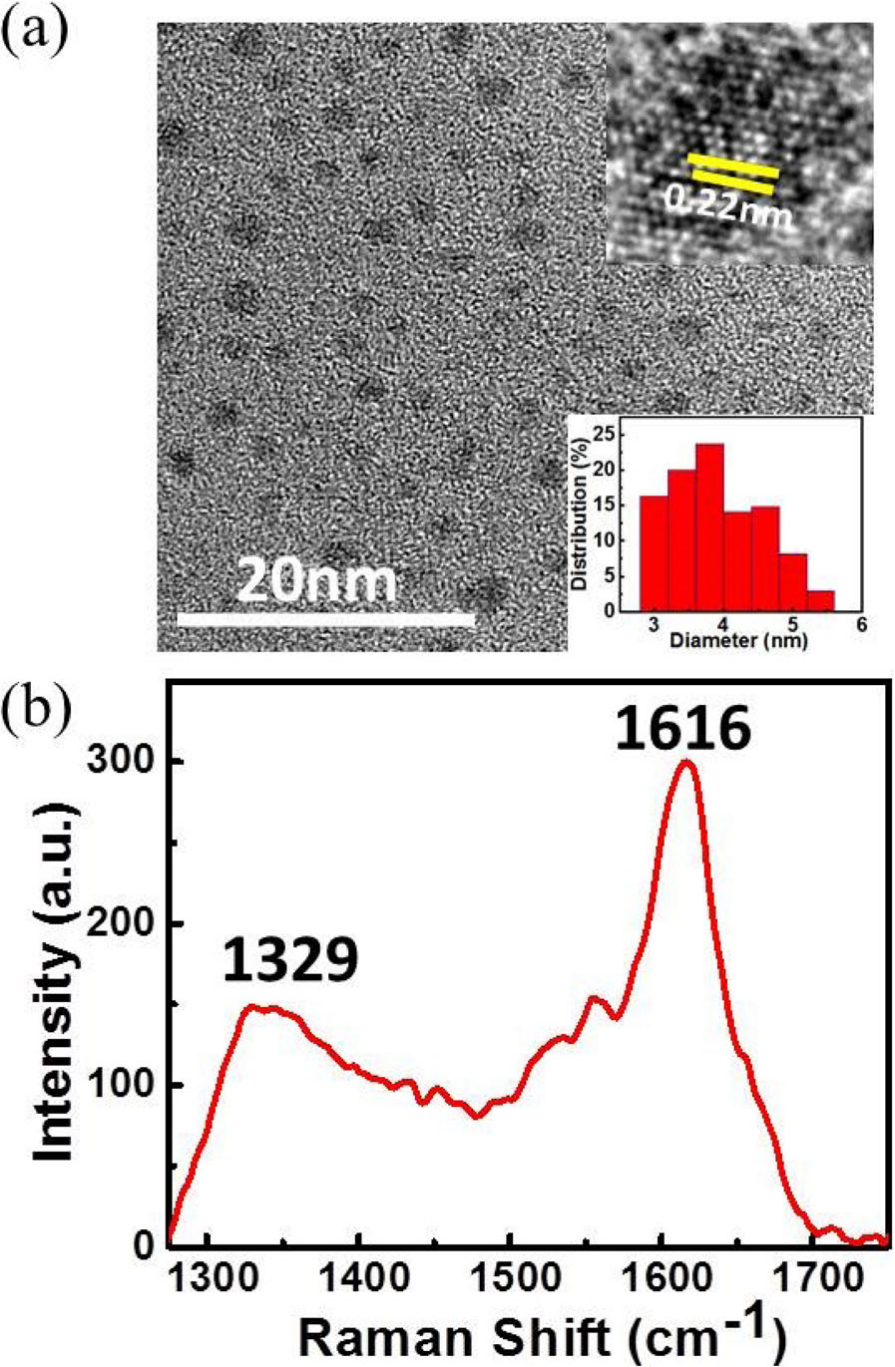
(**a**) TEM image of un-passivated NDs: the top inset corresponds to the HRTEM image of NDs, and the bottom inset corresponds to their size distribution of NDs. (**b**) Raman spectrum of un-passivated NDs

The morphology, size distribution, and lattice structure of un-passivated NDs are shown in Fig. 2a. It can be seen from the TEM image that the prepared ND particles are well-dispersed, and their shape is almost spherical. The inset HRTEM image in Fig. 2a shows that the spacing of the crystal face is approximately 0.22 nm which corresponds to the (111) plane of a diamond. The size analysis is based on 100 particles, as shown in the inset of Fig. 2a, and the average size of NDs is approximately 3.8 nm. Raman spectroscopy was performed to further prove that the sample exhibits a diamond phase. As shown in Fig. 2b, two peaks are located at 1329 cm^−1^ and 1616 cm^−1^ and are labeled peak D and peak G, respectively. Peak D originates from the zone center mode of *T*_*2g*_ symmetry of sp^3^ fraction [25]. Peak G is the zone center mode of *E*_*2g*_ symmetry of sp^2^ fraction [26, 27], and this indicates that defects exist in the NDs. When compared with the intrinsic peak of a diamond at 1330 cm^−1^, peak D downshifts and broadens. This is because the size of NDs is smaller than the Bohr radius of diamond (6 nm) [28], which results in a quantum confinement effect [29]. As shown by Raman results, the intensity of peak G is stronger than that of peak D. However, the scattering cross-section of sp^2^ is 50–230 times larger than that of sp^3^, and thus, the Raman scattering of sp^2^ is significantly more sensitive than that of sp^3^ [30]. Therefore, it can be concluded that most of the products are NDs by considering the results of TEM and Raman scattering.

**Fig. 3:**
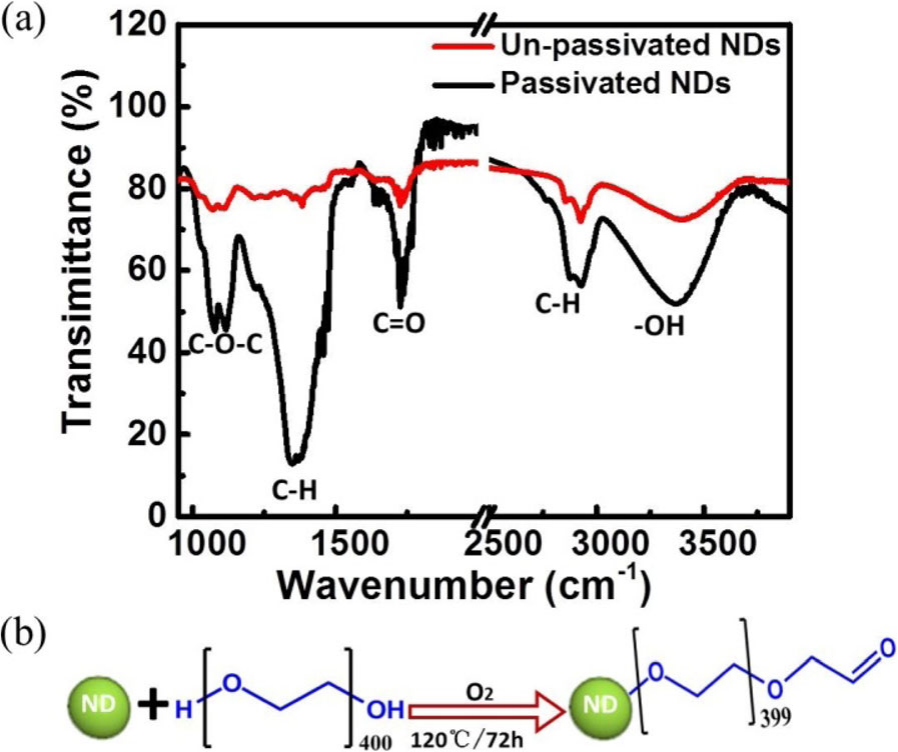
(**a**) FTIR spectra of un-passivated and passivated NDs. (**b**) Schematic of surface oxidation during passivation

In order to investigate NDs’ surfaces, FTIR spectra were studied. As shown in Fig. 3a, for either un-passivated or passivated NDs, the spectra exhibit a strong and broad peak at 3380 cm^−1^ that is caused by stretching vibration of –OH bonds. The absorption peaks located between 2800 and 3000 cm^−1^ originate from the stretching vibration of C–H bonds while the 1380 cm^−1^ peak is due to the in-plane bending vibration of C–H bonds [31]. Given that NDs are prepared in ethanol, some –OH and –CH_*x*_ bonds are adsorbed on the surface of NDs during the preparation process. Additionally, certain oxidation results from the presence of oxygen in the ethanol solution, thereby resulting in a small amount of C=O and C–O–C bonds generated on the surface of un-passivated NDs, and this is clearly observed in the spectrum. One of the bonds is located at 1726 cm^−1^ and is induced by the stretching vibration of C=O bonds [20]. The other bonds correspond to a symmetric bimodal at 1074 cm^−1^ and 1113 cm^−1^, which originate from the C–O–C bonds [32]. However, after passivation by PEG_400N_, the intensity of all absorption peaks increases considerably. In addition to the massive introduction of C–H and –OH bonds, the oxidation degree significantly improved, which implies that a large number of the C=O and C–O–C bonds are formed on the passivated NDs’ surface. At 120 °C, high pressure is formed inside the reactor with ethanol. Given the presence of oxygen, the hydroxyl groups on the surface of NDs are oxidized to form C=O and C–O–C bonds, as shown in Fig. 3b. Therefore, it is proven that NDs are successfully passivated by PEG_400N_.

**Fig. 4:**
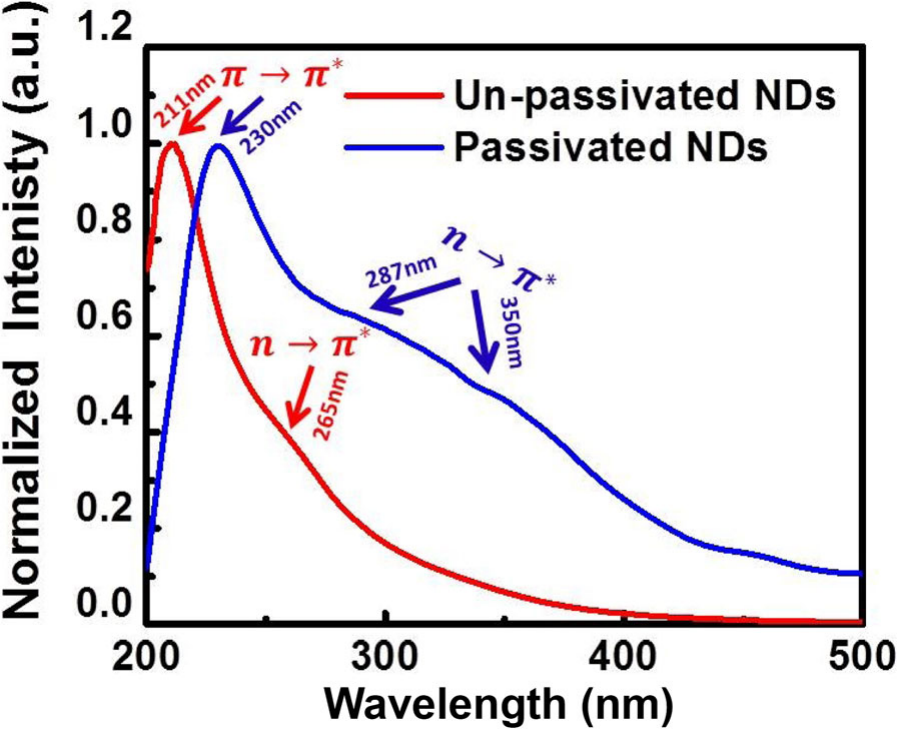
UV-Vis absorption spectra of un-passivated and passivated NDs

Figure 4 shows the UV-Vis absorption spectra of un-passivated and passivated NDs. The absorption spectra of un-passivated NDs exhibit a steep peak at 211 nm originating from *π* → *π*^∗^ transition of C=C bonds and a shoulder at around 265 nm originating from *n* → *π*^∗^ transition of C=O bonds of NDs [33]. Compared with un-passivated NDs, the passivated NDs display a shift in *π* → *π*^∗^ transition to 230 nm, and the 19-nm redshift is potentially due to the narrowing of NDs’ bandwidth given the quantum confinement effect [34], which is induced by the increase in size after passivation [35]. Furthermore, the passivated NDs exhibit two shoulders at 287 nm and 350 nm both from *n* → *π*^∗^ transition and significantly exceed that of un-passivated NDs. The evident *π* → *π*^∗^ *and n* → *π*^∗^ transitions indicate that there are highly localized *π* states, and different trapping states are formed due to defects caused by surface oxidation.

**Fig. 5:**
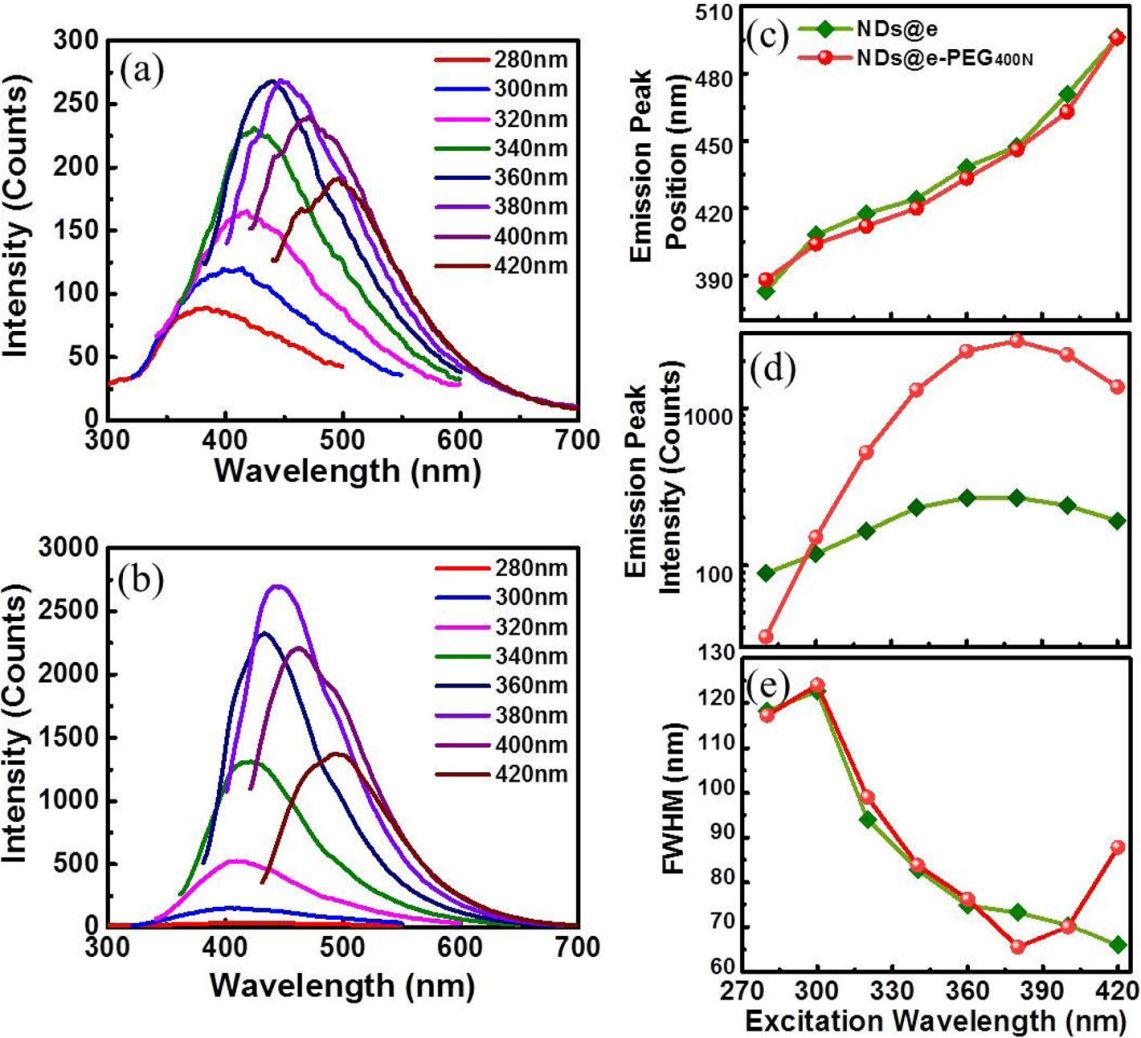
Luminescence spectra of un-passivated (**a**) and passivated (**b**) NDs excited at 280–420 nm. The change in luminescence peak wavelengths (**c**), luminescence peak intensity (**d**), and FWHM (**e**) are various with the excitation wavelength

The luminescence spectra of NDs are shown in Fig. 5. Figure 5a shows un-passivated NDs’ maximal luminescence at 440 nm and 447 nm, which are nearly equal under 360 nm or 380 nm excitation. For passivated NDs, the maximal luminescence peak at 440 nm is excited by 380 nm wavelength, as shown in Fig. 5b. After comparison, the results indicate that under the same conditions, the luminescence peak positions of un-passivated and passivated NDs are almost unanimously dependent on the excitation wavelengths. When the excitation wavelength changes from 280 to 420 nm, the luminescence peaks redshift from 380 to 495 nm as shown in Fig. 5c. As shown in Fig. 5d, the luminescence intensity increases and then decreases with increasing excitation wavelength for either sample. The difference is that the intensity of passivated NDs always exceeds that of un-passivated NDs. The ratio of luminescence peak intensity (*I*_passivated_/*I*_un-passivated_) reaches 10 when excited at 380 nm. As shown in Fig. 5e, the full width at half maximum (FWHM) of the luminescence displays an overall decreasing trend, and the trend is almost consistent for both samples when the excitation wavelength is less than 360 nm. The FWHM began to diverge when the excitation wavelength corresponds to 380 nm. For un-passivated NDs, their FWHM gradually decreases, and it reaches a minimum of 63 nm for passivated NDs when the excitation wavelength is 380 nm. As shown in Fig. 5d, the enhancement due to surface passivation is not equal and it reaches the maximum with excitation at 380 nm, and the reason may be surface passivation makes NDs distribute more uniformly, thereby FWHM decreases. Thus, the FWHM relatively increases due to more discrete NDs’ luminescence with excitation at 400 nm and 420 nm.

**Fig. 6:**
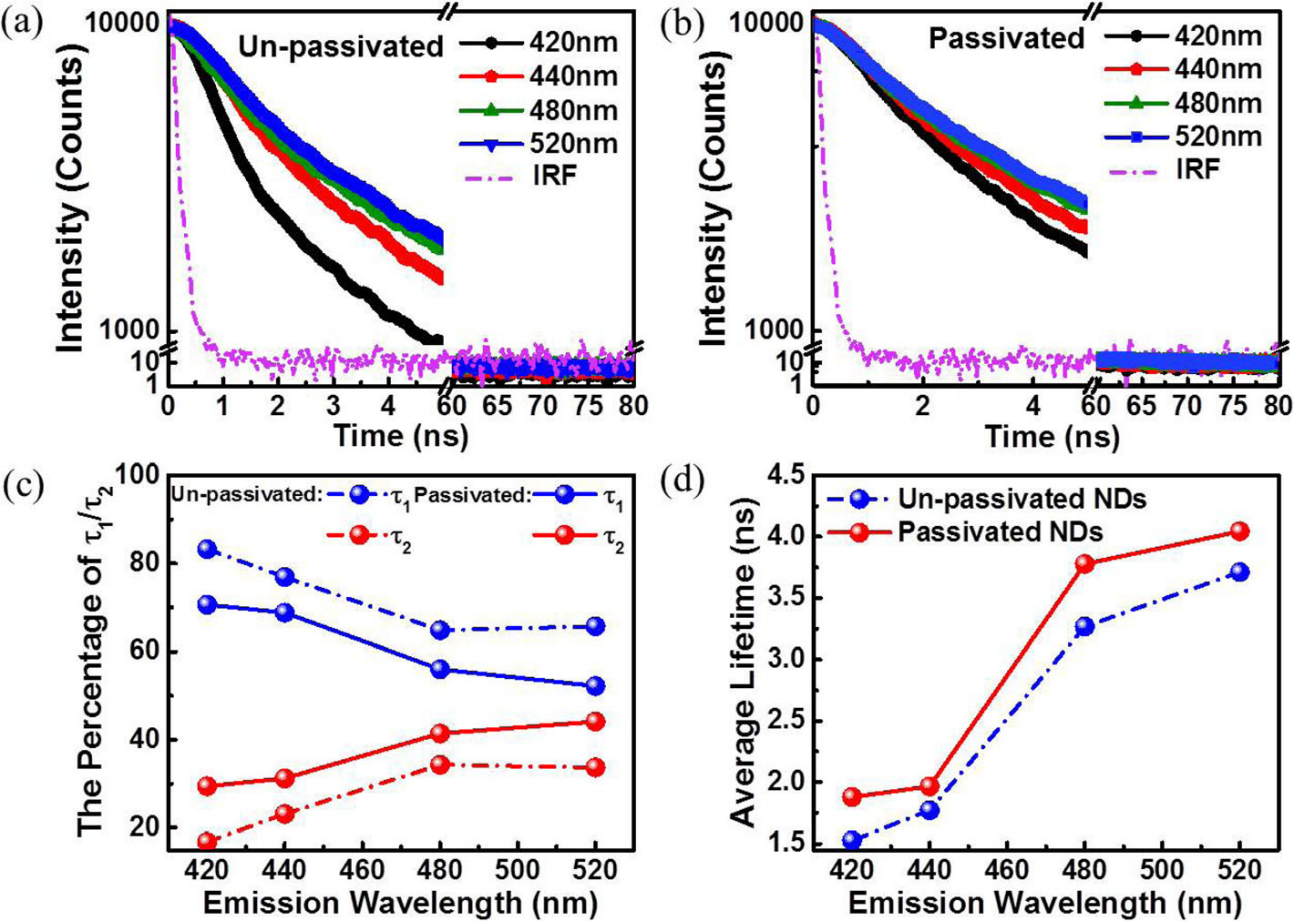
Time-resolved luminescence of un-passivated (**a**) and passivated (**b**) NDs at different luminescence wavelengths. (**c**) Percentage of *τ*_1_ and *τ*_2_ changes at different luminescence wavelengths. (**d**) Average lifetime of different luminescence wavelength for passivated and un-passivated NDs

In order to investigate the luminescence mechanism of NDs with different surface states, transient-state spectra are obtained using the time-resolved method, and the decay curves are shown in Fig. 6a, b. Evidently, the luminescence decay rate of un-passivated NDs is faster than that of passivated NDs. The decay curves are fitted with three exponential functions and tabulated in Table 1. At 420 nm and 440 nm, decay data can be well fitted with components *τ*_1_ and *τ*_2_. At 480 nm and 520 nm, they can be well fitted with three components *τ*_1_, *τ*_2_, and *τ*_3_. Specifically, *τ*_1_ (1.00–1.91 ns) corresponds to the fast component, which is correlated with the non-radiative recombination of electron trapping during transition. Furthermore, *τ*_2_ (5.51–6.41 ns) and *τ*_3_ (16.3–32.8 ns) correspond to slow components, which are mainly derived from radiative recombination. Additionally, *τ*_2_ corresponds to the lifetime of radiative combination from trapping states to the localized *π* state, and it remains almost unchanged at 5.51–6.41 ns for NDs. Moreover, *τ*_3_ is attributed to the electron’s radiative transition from a molecule-like cluster [36].

**Table 1:** Lifetimes (*τ*_1_, *τ*_2_, and *τ*_3_) and relative contributions for un-passivated and passivated NDs at 390 nm excitation

	*λ*_Em_ (nm)	*τ*_1_ (ns)	*τ*_1_ (%)	*τ*_2_ (ns)	*τ*_2_ (%)	*τ*_3_ (ns)	*τ*_3_ (%)
Un-passivated NDs	420	1.00	83.2	5.51	16.8	–	–
	440	1.49	76.9	6.15	23.1	–	–
	480	1.50	64.8	5.64	34.3	23.0	0.9
	520	1.62	65.7	6.03	33.7	32.8	0.6
Passivated NDs	420	1.71	70.6	5.82	29.4	–	–
	440	1.91	68.8	6.41	31.2	–	–
	480	1.83	56.0	5.88	41.4	17.6	2.6
	520	1.75	52.2	5.86	44.1	16.3	3.7

For un-passivated NDs, *τ*_1_ (1.00 ns) accounts for 83.2% at 420 nm. When the luminescence wavelength increases to 520 nm, *τ*_1_ reaches to 1.62 ns while the proportion decreases to 65.7%. The reason is attributed to the increased deviation between the trapping state and excited state at the band edge. The *τ*_2_ component is maintained at approximately 6.0 ns, although its proportion increased from 16.8% (420 nm) to 33.7% (520 nm). It should be noted that at 480 nm and 520 nm, slow component *τ*_3_ is present, but the proportion is less than 1%. For passivated NDs, the longest *τ*_1_ and *τ*_2_ appear at the strongest luminescence at 440 nm and correspond to 1.91 ns and 6.41 ns, respectively. The lifetimes of both components decrease when the luminescence peak moves toward the band edge. The contribution of fast component *τ*_1_ gradually decreases from 70.6 to 52.2%, the slow component *τ*_2_ increases from 29.4 to 44.1%, and *τ*_3_ increases from 2.6 to 3.7%. It is worth noting that the fast component is the main contributor to the luminescence lifetime of NDs.

The proportions of lifetimes *τ*_1_ and *τ*_2_ for un-passivated and passivated NDs are shown in Fig. 6c. Generally, the fast component *τ*_1_ exhibits an overall decrease after NDs are passivated while slow components *τ*_2_ and *τ*_3_ display opposite trends. This is because the trapping states are modified, which leads to more possibilities for radiative combination from trapping states to *π* state. Inevitably, the decrease in the fast component and the increase in slow components increase the average lifetime of passivated NDs as shown in Fig. 6d. Hence, the luminescence decay rate of passivated NDs is slower than that of un-passivated NDs. Furthermore, for either un-passivated or passivated NDs, the average lifetime gets longer with increases in luminescence wavelength. Electrons tend to transfer from trapping states with lower energy levels to the *π* state to realize radiative recombination, and thus, more slow components contribute to the lifetime of longer luminescence wavelengths.

## Conclusions

In conclusion, well-dispersed NDs with dominant sp^3^ phase and an average size of 3.8 nm were successfully prepared in this study. The NDs are passivated by PEG_400N_ for future applications. After passivation, three absorption bands appeared in the absorption spectra. One of the bonds is located at 230 nm originating from *π* → *π*^∗^ transition and displays a 19-nm redshift when compared with un-passivated NDs. The luminescence peaks of NDs were almost unchanged with or without passivation, and NDs emit significantly blue luminescence at 440 nm after passivation. Based on time-resolved luminescence analysis, passivated NDs decay slower than un-passivated NDs. This is due to the increased proportion of radiative combination from trapping states to localized *π* states transition due to surface modification. The results indicate that passivation does not destroy the structure of NDs, and instead improves their luminescence intensity as high as 10 times and prolongs the lifetime, which is very important for application in target tracking and localization in vivo.

## Data Availability

The authors declare that the materials, data, and associated protocols are available to the readers.

## Abbreviations

NDs: Nano-diamonds
fs-PLAL: Femtosecond pulsed laser ablation in liquid
FTIR: Fourier transform infrared spectroscopy
UV-Vis: Ultraviolet-visible
FWHM: Full width at half maximum
TEM: Transmission electron microscope
HRTEM: High-resolution transmission electron microscope
PEG_400N_: Polyethylene glycol-400N
